# Long-term survival of a patient with an inoperable thymic neuroendocrine tumor stage IIIa under sole treatment with *Viscum album* extract: A CARE compliant clinical case report

**DOI:** 10.1097/MD.0000000000018990

**Published:** 2020-01-31

**Authors:** María Reynel, Yván Villegas, Paul G. Werthmann, Helmut Kiene, Gunver S. Kienle

**Affiliations:** a Centro Médico Antroposófico, Lima, Peru; b Institute for Applied Epistemology and Medical Methodology at the University of Witten/Herdecke; c Center for Complementary Medicine, Institute for Infection Prevention and Hospital Epidemiology, Medical Center—University of Freiburg, Faculty of Medicine, University of Freiburg, Freiburg im Breisgau, Germany.

**Keywords:** carcinoid tumor, neuroendocrine tumors, superior vena cava syndrome, thymus neoplasms, *Viscum album*

## Abstract

**Rationale:**

Thymic neuroendocrine tumor (TNET) is very rare and characterized by a tendency to invade adjacent structures, frequent metastasis, resistance to therapy, and a poor prognosis. *Viscum album* extracts (VAE) have shown immunological, apoptogenic, and cytotoxic properties.

**Patient concerns:**

A 54-year-old Peruvian man was suffering from constant fatigue, cough, dyspnea, and fever for a couple of months.

**Diagnoses:**

He was diagnosed with TNET (12.8 cm × 10 cm × 7 cm) stage IIIa, G1. Due to the size and extensive invasiveness (vena cava superior, also obstructing 85% of its lumen, pericardium, and pleura), the TNET was inoperable.

**Interventions:**

We report the case of this patient who declined chemotherapy and was treated instead with sole subcutaneous VAE 3 times per week for 85 months. No other tumor-specific intervention was applied.

**Outcomes:**

Quality of life (QoL) improved substantially. The patient returned to work, and the tumor remained stable for 71 months. Thereafter, the tumor progressed, and the patient died 90 months after initial diagnosis. Besides self-limited local skin reactions around the application site, no side effects occurred.

**Lessons:**

This is an exceptionally good course of disease of an inoperable, large, obstructing, and invasive TNET with a reduced baseline condition (Karnofsky index: 50–60) due to pronounced symptoms. Given the considerable reduction of symptoms and improved QoL following the onset of VAE therapy and other reports describing long disease stability and improvement of the QoL using VAE in different cancer types, we presume that the VAE treatment was supportive in this case. As TNETs are rare and few trials are available, future treatments of TNETs using VAE should be carefully documented and published to help determine whether further investigation of the use of VAE in TNET treatment is worthwhile.

## 1 Introduction

Thymic neuroendocrine tumor (TNET) is an exceedingly uncommon primary thymic neoplasm with neuroendocrine differentiation [5% of all thymic tumors and 0.4% of all neuroendocrine tumors (NETs); 0.2 cases per million in the United States] that generally presents as a mass within the anterior mediastinum.^[1,2]^ The World Health Organization classification from 2015 subdivides TNET into 3 groups: low grade (20%, typical carcinoid), intermediate grade (40%–50%, atypical carcinoid), and high grade (15%–25%, large cell neuroendocrine carcinoma and small cell carcinoma).^[3]^ TNET is generally characterized by relatively aggressive clinical behavior, a tendency to invade adjacent structures (mediastinal fatty tissue, lung, pericardium, great vessels), frequent metastasis, resistance to therapy, and a prognosis that is generally inferior to other NET of similar stages.^[2]^ It usually occurs in middle-aged males (median age: 54 years; male-to-female ratio: 3:1).^[2,4]^ Clinically, patients may be asymptomatic or have signs and symptoms due to the compression or invasion of mediastinal structures (ie, cough, chest pain, and superior vena cava [SVC] syndrome).^[5]^

Given the small number of reports and case series and the absence of prospective trials due to the rarity of this tumor type, there are no uniform treatment strategies. However, surgery is the mainstay of therapy for resectable cases. Patients with operable TNET live significantly longer than those with an inoperable tumor (median 109 months vs 46 months, respectively).^[6]^ The median survival for patients with localized, regional (like the patient described here), and distant metastases are 110 months, 59 months, and 35 months, respectively.^[2]^ Involvement of great vessels and presence of an SVC syndrome indicate poor prognosis (SVC syndrome was associated with a mean 6-month survival, investigated in different tumor types).^[7–9]^ The evidence supporting a benefit for the adjuvant or definite radiotherapy or chemotherapy is extremely limited. Still, radiotherapy is partly suggested, as well as fluorouracil, capecitabine, and cisplatin/carboplatin plus etoposide.

*Viscum album* L. (European mistletoe) is a hemiparasitic shrub that grows on different host trees (ash, birch, apple, oak, and others) and contains a variety of bioactive compounds; the most studied compounds are mistletoe lectins (ML) and viscotoxins.^[10,11]^*V album* extracts (VAE) from the ash host tree (VAE fraxini) show the highest concentrations of ML compared to VAE from other host trees. In particular, the MLs in VAE demonstrate antineoplastic activity, strong cytotoxic and apoptosis-inducing effects, immune stimulation, inhibition of tumor cell migration, and neoangiogenesis.^[11–15]^ Various injectable standardized VAE preparations are commercially available and are widely used among cancer patients in German-speaking countries.^[11]^ Clinical trials and other studies have documented an improvement of the quality of life (QoL) and potential effects on survival.^[16–18]^ Currently, clinical trials using VAE preparations in different types of tumors are being conducted in Sweden (NCT02948309), the United States (NCT03051477), and sites in Germany and Egypt (NCT02106572). However, to the best of our knowledge, no clinical studies have been published on the use of VAE in TNET. This case report was prepared following the CARE guidelines.^[19]^

## 2 Case presentation

**Figure 1:**
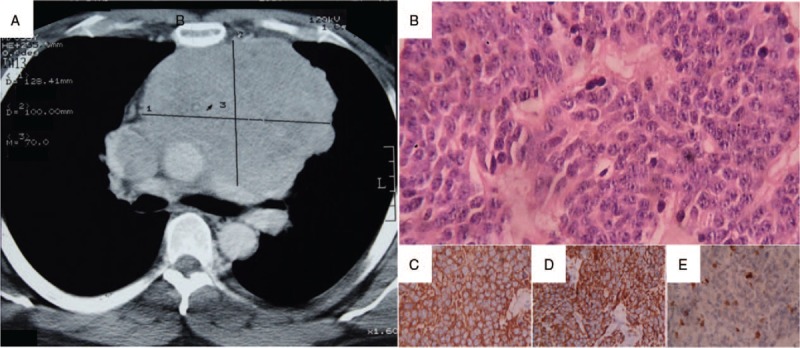
(A) Chest computed tomography scan of the initial diagnosis: anterior mediastinal tumor 12.8 cm × 10 cm × 7 cm. (B–E) Biopsy of the tumor (hematoxylin and eosin stain 400×). (B) Organoid nests composed of uniform polygonal cells with centrally located nuclei and finely granular nuclear chromatin forming rosettes. On immunohistochemistry, tumor cells are positive for (C) synaptophysin and (D) cytokeratin; (E) Ki-67 10% to 15%.

A 54-year-old Peruvian man was suffering from constant fatigue, cough, dyspnea, and fever for a couple of months. In emergency service, a computed tomography (CT) scan revealed an anterosuperior mediastinal tumor (12.8 cm × 10 cm × 7 cm) and pericardial effusion (Fig. 1). He underwent an exploratory sternotomy, where it was found that the tumor with a cerebroid aspect occupied two-thirds of the anterior mediastinum and infiltrated the pericardial cavity and the right pleura (T3N0M0, G1, stage IIIa). Surgical resection was not feasible. However, a pericardiocentesis (800 cc, hematic) and biopsy were performed. The pathology report described it as a thymic NET consistent with a typical carcinoid (histological criteria: <2 mitoses/2 mm^2^; no necrosis; Fig. 1). The diagnosis was corroborated with immunohistochemistry markers (positivity of synaptophysin and cytokeratin; negativity of TTF1 and Ki-67 10%–15%). Given the rarity of this type of tumor, the patient was referred to the best-specialized cancer center in Peru (Instituto Nacional de Enfermedades Neoplásicas). A second CT scan was performed, revealing that the tumor was in intimate contact with great vessels and was infiltrating the SVC with 85% of its lumen obstructed. This obstruction corresponded with the symptoms that the patient suffered from the beginning (SVC syndrome). The patient underwent a second surgery, where again the resection was not possible. The pathology report confirmed the previous diagnosis.

**Figure 2:**
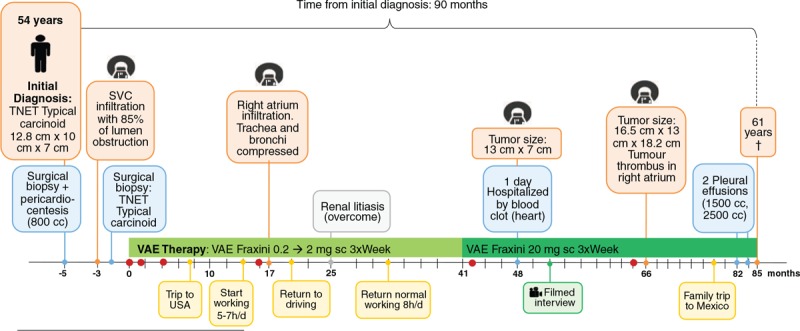
Timeline of the patient with invasive mediastinal neuroendocrine tumor.

Chemotherapy was suggested; however, the patient declined it because of the narrow range of effectiveness explained by his oncologist. The patient decided to look for a different treatment with an integrative approach. Two months later, he presented himself at our institution [Centro Médico Antroposófico (CMA)]. An integrative treatment with subcutaneous VAE applications from the ash host tree (AbnobaVISCUM Fraxini) was suggested in increasing doses of 3 times per week. The patient started with an application of 0.2 mg VAE fraxini into the upper arm; after 2 weeks, the dose was increased to 2 mg and was maintained for the following 40 months. Then the dose was increased to 20 mg and continued at this dosage for another 44 months until the patient's death (for details of the course, see Fig. 2).

Subcutaneous VAE injections induced self-limited local erythema and swelling of the skin around the application site up to a maximum diameter of 5 cm, which was tolerable to the patient. These reactions are expected during the therapy with VAE, as they indicate an immune stimulation and can be used for dose adaptation.^[20]^

**Figure 3:**
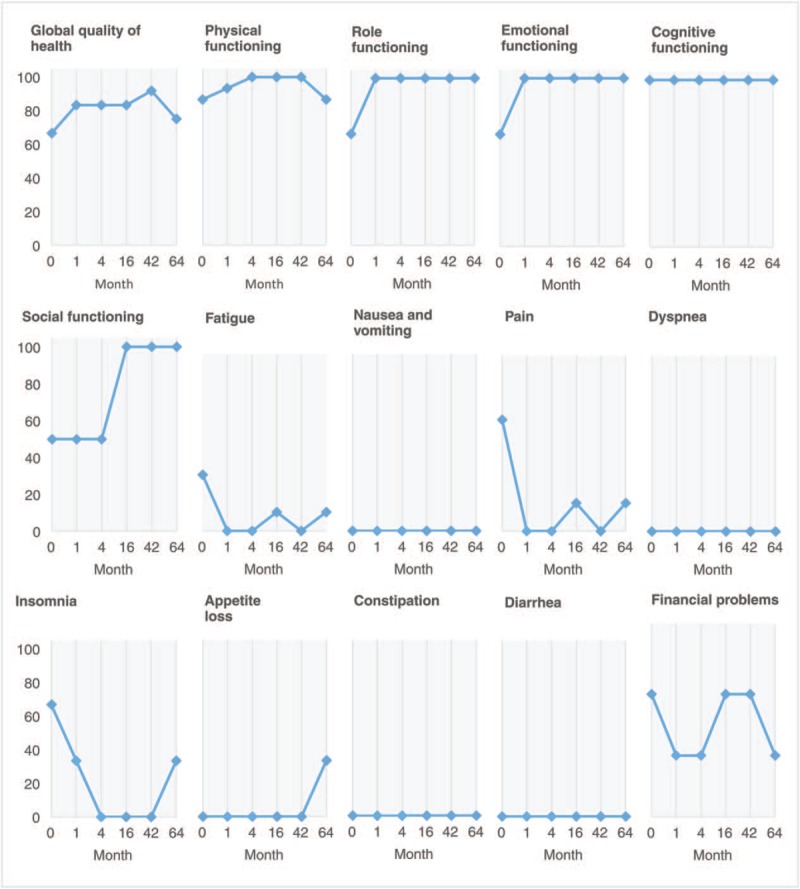
Changes on the 15 quality-of-life scales of the European Organisation for Research and Treatment of Cancer Quality of Life questionnaire (EORTC-QLQ-C30) of the patient. Scale from 0 (not at all) to 100 (very much). Month 0 represents the beginning of *Viscum album* extract therapy.

Once the VAE therapy started, the patient experienced continuous improvement in his QoL. At the beginning of the treatment, the patient suffered from pain, fatigue, and insomnia, all of which continuously improved during the VAE therapy. Also, the functional scales of his QoL questionnaire (EORTC-QLQ-C30)^[21,22]^ improved substantially; the other symptoms were tolerable (for details of the course, see Fig. 3). These improvements made it possible for the patient to progressively return to regular working hours, drive his car again, and travel to visit his family. Follow-up CT scans showed disease stability with no signs of nodules or distant metastases for 71 months after his initial diagnosis. The patient's last CT scan (month 66 of VAE treatment) revealed a tumor that had progressed in size (16.5 cm × 13 cm × 18.2 cm) and a tumor thrombus in the right atrium. Despite this, the patient continued to feel good, and 14 months later, he decided to travel to Mexico to attend the wedding of his oldest daughter. After he returned from Mexico (month 78), his condition aggravated. He had peripheral edema, the symptoms of fatigue, and cough returned and steadily increased. The pleural effusions started in month 82 and increased in volume over time (1500–2500 mL). In month 85, 1 month after his last pleural effusion, the patient died at the age of 61 of a heart attack, probably caused by the compression of pericardium due to a pericardial effusion (90 months after initial diagnosis and 85 months after the initiation of VAE therapy).

### 2.1 History, antecedents, and concomitant therapies

The patient was the third-born child of 10 who became an electromechanical engineer and specialized in graphics machines. He had a high-calorie and high-protein diet (red meat, daily soda) and was overweight (99 kg; height 180 cm) and a social smoker. After his diagnosis and with the constant support of his family, he changed to a vegetarian diet and began taking oral food supplements such as vitamin C, vitamin B complex, and *Morinda citrifolia*, and *Uncaria tomentosa* extracts. He also quit smoking. His father had died of a heart attack years before.

He additionally received anthroposophic remedies. From the time of his diagnosis, he took a preparation with *Onopordum acanthium*, *Primula veris*, and *Hyoscyamus niger* (Cardiodoron) 10 drops 3 times daily to protect cardiac function. During his trips, he took a preparation with *Fragaria vesca* L. and *Vitis vinifera* L. (Hepatodoron), 2 pills per day to protect hepatic function.

Further medications were added at month 78 (after his last trip); these included 40 mg furosemide and 25 mg spironolactone (for peripheral edema), 125 μg digoxin, 100 mg atenolol, and 100 mg acetylsalicylic acid (for cardiovascular function).

### 2.2 Patient perspective (from a filmed interview)

In month 52 of treatment, the patient gave his testimony about his experience with mistletoe therapy:

The oncologists tried in 2 surgeries to remove the tumor, but they could not because it affected vital organs. They did not give me an alternative. Chemotherapy had little chance of effectiveness. I had to stop working. I walked 2 blocks and could not walk any more; I felt exhausted. When someone finds out that he has got cancer, I felt fear and became depressed, but when talking with you (CMA), one changes the way of thinking about the disease a lot. I started with mistletoe treatment, and everything turned upside down. My quality of life was excellent I wanted to go back to work immediately, but I was told to go slowly, so I gradually increased the work hours. I started with 4, then 6, then 8 hours a day, and sometimes I would get up to 10 because I felt really good. I have my tumor with me, but I feel pretty good. I live together with my disease.

## 3 Discussion

We present a case of large, invasive, and symptomatic TNET consistent with a typical carcinoid protruding into the SVC (T3N0M0, G1, stage IIIa) treated with only subcutaneous VAE therapy for 85 months. The patient experienced a continuous improvement of QoL (Fig. 3) and 90 months of survival (with the tumor remaining stable for 71 months). No nodules or distant metastases appeared.

In several small studies and case reports, the use of sole VAE has been investigated or reported in the treatment of different types of cancer such as bladder,^[23]^ hepatocellular carcinoma,^[24]^ breast and other gynecological types of cancer,^[25]^ adenoid cystic carcinoma,^[26]^ melanoma,^[27]^ Merkel cell carcinoma,^[28]^ malignant pleural mesothelioma,^[29]^ and primary cutaneous B-cell lymphoma.^[30]^ They mostly described an improvement of QoL, unusually long survival time, and, in some cases, a tumor regression.

The main symptomatology of the patient after the initial diagnosis was due to the SVC syndrome caused by direct invasion of the tumor into the SVC (85% of blood flow obstruction). The reduction of fatigue, pain, and insomnia during VAE therapy was a crucial factor for the patient to resume his normal activities (regular work hours, driving a car, and trips abroad); this contributed to improving social and emotional functions, also evaluated in the QoL questionnaire. Improvement of QoL during VAE therapy was found in several clinical studies.^[16]^ A confirmative randomized controlled trial in patients with advanced pancreatic cancer found substantial improvements in QoL, including weight gain in the VAE-treated group; this trial also found a statistically significant survival benefit of 2.1 months (4.8 month vs 2.7 months) for the VAE-treated group.^[17,18]^

In retrospective nonrandomized studies, locally invasive TNET had a median survival of 59 months and a 5-year survival rate of 48%; moreover, 46 months of median survival was found when tumor resection was not feasible versus 109 months when it was.^[2,6]^ In 2 large meta-analyses performed by the Mayo Clinic and the European Organisation for Research and Treatment of Cancer, researchers concluded that cancer patients reporting a good QoL have a statistically significant longer overall survival.^[31]^

The reason for increasing the dose of VAE was to enhance the immune system modulation, and thus to support immunologic tumor control.^[32]^ A strong immune response after local high-dose VAE applications has been repeatedly reported.^[26,27,30,33,34]^ Most NET manifest a tumor immunoevasion.^[35]^ De Hosson et al^[36]^ studied the possible mechanisms for the tumor immunoevasion and found nonexpression of programmed death ligand-1 in tumor cells and T-cells corresponding to a cold immune microenvironment in NET; furthermore, indoleamine 2,3-dioxygenase and tryptophan 2,3-dioxygenase expression were found in tumor cells, each of which additionally plays a role in immunosuppression.^[36]^ The high cytotoxic, apoptotic, and, especially in the case presented here, the immunostimulatory and immunomodulatory properties of VAE may have played a role in tumor control, as it is known that VAE treatment enhances immunological recognition and increases immune response against the tumor via a variety of reactions (eg, proliferation and antigen presentation of dendritic cells, proliferation of CD4+ T-cells, increase of natural killer cell-mediated cytotoxicity, and release of cytokines including interleukin 1 and 6).^[14,37]^

Despite the low grade of the TNET, which is associated with a better survival, the patient in this report presented several risk factors that are all associated with a very poor prognosis: the large size of the tumor, the invasion and obtrusion of the vena cava and its symptoms, and the invasion of pericardial and pleural cavity that evidenced its stage IIIa and unresectability (2 attempts of surgical removal). Therefore, the 90 months of survival of this patient is far beyond what can be statistically expected. As no other tumor-specific treatment was used, we presume that VAE contributed to this positive outcome: improvement of QoL, control and relief of symptoms of SVC syndrome, increased the survival time of 90 months after initial diagnosis, and disease stability with no signs of nodules or distant metastases for 66 months of VAE treatment. Still, this article describes only a single case. Considering the current state of evidence, VAE injections cannot replace surgery or other effective anticancer treatments.

## 4 Conclusions

This is the first report on a subcutaneous VAE treatment in a patient with a large, invasive, and symptomatic TNET associated with a substantial QoL and exceptional long disease stability and survival. As TNET is a very rare cancer and clinical trials are challenging to conduct, further cases of VAE use in TNET should be carefully documented and published to help determine whether further investigation of the use of VAE in TNET treatment is worthwhile.

## Acknowledgments

We are thankful to the Stiftung Integrative Medizin, Stuttgart, Germany and Christophorus Stiftung, Stuttgart, Germany for financial support. We also thank Dr Yober Espinoza Zárate (CMA) for reviewing the CT scans, Yesenia Silva Mendoza (CMA) for providing histology slides, and Dr Cesar Vela-Velasquez (Instituto de Investigación de Citopatología) for the histological images.

## Author contributions

**Conceptualization:** María Reynel.

**Data curation:** María Reynel, Yván Villegas.

**Funding acquisition:** Helmut Kiene, Gunver S. Kienle.

**Investigation:** María Reynel.

**Methodology:** Paul G. Werthmann, Helmut Kiene, Gunver S. Kienle.

**Supervision:** Yván Villegas, Paul G. Werthmann, Helmut Kiene, Gunver S. Kienle.

**Validation:** Yván Villegas, Paul G. Werthmann, Helmut Kiene, Gunver S. Kienle.

**Visualization:** María Reynel, Paul G. Werthmann, Helmut Kiene, Gunver S. Kienle.

**Writing – original draft:** María Reynel.

**Writing – review & editing:** Yván Villegas, Paul G. Werthmann, Helmut Kiene, Gunver S. Kienle.

María Reynel orcid: 0000-0002-3899-9708.
